# Dental Jurisprudence

**Published:** 1900-03

**Authors:** F. F. Graves

**Affiliations:** Denver, Colo.


AMERICAN JOURNAL

—OF—

DENTAL SCIENCE.

VOL. XXXIII. BALTIMORE, MARCH, 1900. NO. 11.

### Selections.

ARTICLE I.

### DENTAL JURISPRUDENCE,

F. F. GRAVES, D. D. S., DENVER, COLO.

Read before the Colorado Dental Association.

When your honorable President called on me to prepare a paper for your consideration, "Dental Jurisprudence" at once suggested itself to me as a subject that had not been used at your annual meetings, and would probably interest you in a different manner and on a different line of thought from that which is usually employed at the dental conventions.

I shall abbreviate my subject considerably, but will try and touch a little on all the material points.

Dental jurisprudence is defined as that science which teaches the application of every branch of dental knowledge to the purposes of the law; therefore, to understand the subject thoroughly, one would have to possess a complete knowledge of dentistry in all branches, and to know the law sufficiently to intelligently recognize what its requirements may be in a certain case, and be able to state it to a court and jury.

The range of dental jurisprudence must of necessity be limited, but a dental jurist must possess knowledge thorough and complete within its scope.

A court and jury to arrive at a definite and proper conclusion in a case involving any or all branches of dentistry, must from necessity turn to some member of the dental profession to have these points made clear, and technical terms and phrases interpreted which are obscure in meaning to the unlearned or layman, but which can be made perfectly clear by an intelligent dentist in the capacity of an expert.

Now a dentist may be called to testify either as an expert or non-expert. As experts, dentists are classed with M. D.'s and other expert witnesses. An expert witness is any person who, owing to superior knowledge and experience, is qualified to give an opinion superior to the ordinary witness on that branch of science concerning which he is called to testify, and as such, cannot be asked but hypothetical questions, and therefore need not be conversant with the facts in the case at issue. The line between expert and non-expert is always clearly distinguishable. As a rule, non-experts are confined to mere statements of facts, while the expert is summoned to testify or interpret to the satisfaction of the jury, the accepted scientific or technical thoughts upon such questions with which they are unfamiliar.

Before a dentist would be allowed to give expert testimony, he would have to submit to a preliminary examination, or put upon his *voir dire*, which is for the purpose of determining as to his qualifications in that line of knowledge and skill.

He may be called upon to give testimony in all cases originating in the profession, either civil or criminal, to a piece of prosthesis or to malpractice. He may be subpœnaed by either the plaintiff or the defendant, and he is not responsible for the result of his opinions.

Identification by means of the teeth has been employed for a period of a century and a half past; as far back as the middle of the eighteenth century cases are on record where dead and unknown bodies have been positively identified by peculiarities of the teeth when all other means have failed. So, also, have murderers been brought to justice by these means; and so have innocent persons, accused of grave crimes, established their innocence, where peculiarities of the teeth are considered a reliable means of identification in the description of the alleged criminal. Thus where the identity of a corpse is suspected, especially as the hair, clothing, etc., are not sufficient, the dentist of the person whom the deceased is suspected to be, is summoned to examine the mouth of the corpse, and to testify whether the peculiarities of the teeth, etc., are similar to those of his former patient, and thus establish his identity. The dentist has many means of positive identification, such as the presence or absence of teeth, the presence of dentures, fillings, crowns, etc. In this manner, in the case of Timothy Monroe being identified as William Morgan, the murdered Freemason, in 1827. Many of you present have probably read or heard of this case, especially of the disappearance of Morgan, who had written a book exposing the secrets of the society of Freemasons. At any rate, Morgan disappeared, and many theories were advanced as to what had become of him. Finally a body was found that answered almost perfectly to the published description of Morgan. The testimony of Mrs. Morgan and others seemed to leave no doubt but that the body was that of Morgan; the gray beard, the bald head, much hair on the breast, long white hairs in the ears; also a mark of innoculation on the arm, teeth double all the way round, two of them being extracted on the same side of the face, the dentist having the same teeth which fitted exactly, the marks of a surgical operation on the large toe of the left foot, all seemed to be proof of a character sufficient to leave little doubt remaining, notwithstanding the clothes found on the body were different from any Morgan had ever worn. The verdict was that the body was that of William Morgan. Soon afterward a Canadian advertised for the body of Timothy Monroe, which caused the body to be exhumed, when, on the holding of a second inquest, it was discovered that the teeth were not double all around in front and that five teeth had been extracted from this body, while Morgan had lost but two. The second jury found the body to be that of Timothy Monroe.

Time and space will not admit of the citation of any more cases at length. The celebrated case of Tascott, the murderer of Snell, the Chicago millionaire, which filled the papers a few years ago, is another case in point. A man in Philadelphia was arrested for Tascott, and came very near being convicted, when it was discovered that the accused had not the gold fillings in the incisors that Tascott had, and the man's innocence was established primarily by his teeth, corroborated by some other proof. Now we leave the subject of experts and take up malpractice.

Before leaving the subject of experts, however, I want to repeat a little story I heard with reference to a medical expert, and which serves to illustrate that one must not be too technical with his terms in testifying in his case. The story runs, that on a trial for assault and battery, a surgeon in giving his evidence, informed the court that on examining the prosecutor he found him suffering from a severe contusion of the integuments under the left orbit, with great extravasation of blood and ecchymosis in the surrounding tissue, which was in a state of tumefaction; there was considerable abrasion of the cuticle. Judge. "You mean, I suppose, that the man had a black eye?" Witness. "Yes."

It would be easy to multiply examples of this kind. This is not science but pedantry, and serves only to disgust the counsel and judge and bewilder the jury. An expert should, when talking to the layman or unlearned in the profession, use as plain language as is consistent with the case at hand, and so, also, I think the dentist should, in his office.

Malpractice is divided into *negligent*, which embraces those cases where there is no criminal intent or purpose, but gross negligence in bestowing that attention which the case requires (as in a state of intoxication); *ignorant*, where operations and treatments are performed in a way calculated to do injury, and actually does harm, and which a properly educated and skilled dentist would know was not proper in the case, and *willful* malpractice, where a dentist performs operations or treatments with malicious intent to do injury.

Dentists, by holding themselves out to the world as such, engage that they possess the reasonable and ordinary qualifications of their profession, are bound to exercise reasonable and ordinary care, skill and diligence, but that is the extent of their liability. The burden of proof lies with the plaintiff in all actions for malpractice, to show that there was a want of due care, skill and diligence, and that the injury was for want of such skill, care and diligence.

The reasonable and ordinary care and skill which the law requires of dental surgeons is such as those in the same general line of practice and in the same general locality have and exercise in like cases.

Now, in reference to locality, a dentist in a small town or sparsely settled country would scarcely be expected to posses the same skill and knowledge as he who resides in a large city, and can take advantage of the many opportunities afforded him, and it would be unjust for a jury to render a verdict on that line, and the court would, in its instructions, emphasize the fact.

The science of dentistry is constantly changing and advancing. The discovery and introduction of new medicines for treatments and the inventions which are coming into use and being recommended by the majority of dentists who are up to date, as a natural sequence varies the standard of ordinary knowledge and skill; old methods of operating and treatment are being superseded by modern methods. When such changes of treatment and operating are generally known and adopted in a locality they become ordinary, and dental practitioners of such localities are responsible for a knowledge of these changes, and are expected to practice them. There is no arbitrary rule that governs, however; for instance: There may be two or more remedies for treatment of the same case; there may be different instruments for performing the same operation; in such cases the dentist will have to rely on his own judgment, and where reasonable doubt exists he is not liable for adverse results, providing it can be shown that he did the best he could under the circumstances. A dentist must exercise good judgment in performing a new operation, or in the use of a new instrument, and see that no injury follows to the patient as a result of the experiment; for if injury should follow he can be held liable for damages in the same. To illustrate the point, I will, with your permission, cite a case on record of two surgeons who used a new instrument on a patient with a fractured limb, and an action was instituted for malpractice, and the gist of the ruling of the court is as follows: "That it appeared to the court that it was the first experiment with the new instrument; if it was it was a rash action, and he who acts rashly acts ignorantly, and though the defendants may be as skillful in their respective professions as any two gentlemen in the country, yet the court cannot refrain from saying that in this particular case they acted ignorantly and contrary to known rules and usages of surgeons." However, if a dental practitioner has a new method of operating or treatment which appears to him to be applicable to the case, and it is founded on good judgment, and furthermore, he could show to the satisfaction of the court and jury that it was an improvement over the old method in use, or that it was the only one available, he is privileged to use the same, and he would be exonerated if the injury could not be directly attributed to an unskilled or imprudent operation. The aim of the law is to arrive at a just and equitable judgment in the case, as based on the facts presented.

As has been stated, new operations and instruments are added day by day to the science of dentistry. Those that have been adopted and adjudged by the body of the profession necessary for the benefit and advancement of mankind, the dentist is expected to employ; at least, that is what the community and his patients expect of him, viz., that he gave the best services and the benefit of any new discovery in dental science. It has been a question among dentists at different times as to their legal right to give anæsthetics. There can be no question but that the dentist has the absolute right to administer any and all anæsthetics, providing that he complies with all the laws and enactments regulating the practice of dentistry in the state or county in which he resides. He is expected to render the patient the best service and treatment within the limits of his ability, and where in an operation he can mitigate or reduce the pain, he should do so, if the operation demands it, even to the administration of an anæsthetic. He however, must thoroughly understand all of the practical details of the operation which the court would hold to constitute ordinary skill. If he can prove that he is thoroughly familiar with the methods of administering anæsthetics, with all the precautions to be observed, he will not be held liable for adverse results if he applied his knowledge properly to the case.

It of course necessarily follows where life is in danger a greater degree of skill is required of the dentist, and what would be ordinary skill in one case would be adjudged *gross* negligence in the other. Here the legal interpretation of "ordinary skill" is held to include all knowledge and experience the profession has attained on the subject.

The standard of skill required may be illustrated in the following case recorded in an Eastern state: The defendants undertook to extract a tooth while the patient was anæsthetized with nitrous oxide gas; in extracting the tooth the forceps split, and part of the tooth lodged in the patient's throat, causing coughing and vomiting for four weeks. Under the circumstances, the court was of the opinion that the defendants were bound to use extraordinary care. They knew that the plaintiff, while under the influence of anæsthetics, had no control of his faculties; that he was powerless to act, and that he was unable to make the slighest effort to protect himself from any probable or possible consequences of the operation that might follow. He was in their charge and under their control to such an extent that they were bound to exercise the highest skill and diligence to avoid every possible danger, for the law implies duties on men according to the circumstances in which they are called to act.

Now as to a specialist. The law recognizes a difference existing between the relative skill and knowledge of a specialist and a non-specialist or general practitioner; in other words, a dentist holding himself out of the world as a specialist in any particular branch, whether mechanical or surgical, or in the administration of anæsthetics, is by law presumed to possess more than the ordinary skill and knowledge of the subject than the general practitioner. He is expected to use the highest skill and knowledge attained by the profession on the subject, and anything short of that would be condemned.

In cases where there is a reasonable doubt as to what treatment should be used, or what dental appliances may be applied, the dentist would not be held liable for adverse results, providing it could be shown that the error was honest and the dentist had used good judgment in the matter, and where uncertainties exist and the dental surgeon pursues a course that would meet the approval of the body of the profession, there need be no fear as to what would be the result before a court or jury.

We next take up the subject of negligence, and I hope you will pardon me if I touch on this in a very brief manner. A definition of negligence which would be recognized in law as negligence, consists of a want of that reasonable care which should be exercised by a person of ordinary prudence under all existing circumstances, in view of the probable danger of the injury, which would mean as applied to the dental profession, that the dentist is expected and should use reasonable care and judgment in all cases he undertakes, and it is held as a contract—an implied one—to do so, whenever he engages to serve a patient.

The courts have divided negligence into three degrees:

Gross negligence, which is the absence of slight care.

Ordinary negligence, which is the absence of ordinary care.

Slight negligence, which is the absence of great care.

There is no fixed and unswerving rule as to what constitutes care and diligence in every case. All the circumstances will have to be weighed and considered. The one aim the law has in these cases, as in fact in all cases, is to be reasonable, and as it is a question of fact for the judge to decide, it would depend on the evidence presented. In many cases the evidence would prove conclusively of carelessness, such, for instance, as extracting a sound tooth for a diseased one, which in all cases would be gross negligence.

I have said enough of negligence to give you an idea as to what the dentist's rights are, and also what the patient may expect, and will now speak of what would be the dentist's greatest defense in all cases mentioned above. In most all actions for malpractice, from whatever cause, contributory negligence is the principle defense, which means where the injury complained of follows in part from the negligence of the patient in following the instructions of the dentist. Say for instance, in the matter of keeping engagements for the treatment of alveolar abscess; if the patient did not follow the directions of the dentist in every particular, and evil results should follow, as a fistulous opening on the external cheek, which, as you know, produces an ugly scar, contributory negligence on the part of patient would be sufficient defense for the dentist, as it can be plainly seen that the burden of responsibility would be shifted from the dentist to the patient, and the dentist ceases to be held liable.

Where negligence is the ground of an action, it devolves upon the patient or plaintiff to trace the fault for his injury to the dentist, and for this purpose he must show the circumstances under which it occurred. If from these circumstances it appears the fault was mutual, or that the injury was the result of contributory negligence imputable to the patient, he has disproved his right to recover. There is a legal maxim that no person shall profit by his own wrong. Along this line the dentist will always have a great advantage over the prosecutor in all actions for malpractice, because it is rarely that patients absolutely follow the dentist's directions in protracted treatments and operations. In civil suits for damage arising from malpractice, the amount to be recovered is largely a question for the jury to decide.

The principle of compensation is the governing principle in the measurement of damages. The bodily pain resulting from the injury is always to be considered in estimating damages. It is a general principle that every defendent shall be held for all those consequences which might have been forseen and expected as a result of his conduct; but not for those which he could not have foreseen, and was therefore under no moral obligation to take into consideration.

I now in a brief manner will take up contracts. Time admits only of definitions.

A contract is an agreement between two or more persons upon sufficient consideration for the doing or not doing of some particular thing. Contract comprises in its full and liberal signification every description of agreement, obligation or legal tie, whereby a party binds himself or becomes bound, expressly or impliedly, to another to pay a sum of money or perform or omit to perform a certain act. Contracts are either express or implied; express, when formally stated or written; implied, when the inference can be drawn that certain things are to be performed from the actions of the parties thereto; contracts between dentist and patient are usually implied, and may be enforced the same as an expressed on. Under the implied contract, the dentist agrees to render his services with at least ordinary skill and suitable to the case about to be performed. The patient who accepts such service promises to pay a sufficient compensation to be named by the dentist. In this particular, I wish to say that the dentist's reputation and large practice would cut some figure in what would be considered sufficient reward as againt a dentist who assumes an obscure position in the profession. The presumption of law is that both dentist and patient will honestly perform the obligations incident to the contract, and the dentist cannot legally, after once undertaking the contract, withdraw without the consent of the patient; neither can the patient withdraw without the consent of the dentist. Fraud on the part of either party to the contract voids it, and if a dentist warrants his work in the whole or in part, the patient can insist on the guarantee being made good. A dentist may offer his assurances that the work is performed with his best skill and knowledge, but he must not guarantee the permanency of his work. And there is no reason why he should; it only furnishes a precedent or bad example, and educates the people to expect something more and lasting from dental work than that which the dentist can always deliver, and when the work fails he is discredited with that patient.

There is a subject I wish to touch on here, and one which is, I take it, a source of considerable annoyance to the dentist, and also one from which he suffers financially, and that is, broken engagements in the office. Every dentist present suffers more or less from this cause. He is apt to pass it over with a mental protest, thinking there is nothing to be done—no recourse—but to swallow his disappointment and look pleasant. Say he has an appointment at 10. About an hour or so after, and when it is nearly time for another patient to appear, the 10 o'clock one will float serenely in and offer as an apology some frivolous excuse, either ignorant of the inconvenience and loss of valuable time to the dentist or, if they do know, exhibiting a selfish disposition to consider no one's convenience but their own. I know from my own experience, whether from a tardy appearance, or whether they do not come at all, I have been rushed the whole day from what might have been accomplished in a leisurely manner and with greater satisfaction to both the patient and myself. Now the dentist has recourse, and that is, to be paid for lost time; and that is what he is morally and legally entitled to. The amount, of course, would be too trivial to invoke the law, but at the same time his cause would be a just one, and would be upheld by the courts. I state this just to show a dentist's legal position in the matter, providing the patient refuses to pay for time of a broken engagement. The law views the acceptance of an appointment by a patient for a specified time as an *implied contract*, thereby holding the patient responsible for such accepted time if the patient voluntarily violates the contract. The rule is reasonable, because the dentist relies on his professional time for his support. We ought to enforce this rule more. With united action in this matter, the public would soon become educated. The dentist has the same remedy at law as do parties in other lines under implied contract; where no price is stipulated he is entitled to recover a reasonable fee commensurate with the amount of time, skill, knowledge, etc., employed. In a general way, a minor cannot be held liable for services for which he contracts unless it can be shown that work was necessary, and was no more expensive than the case indicated, and then it would be void if the minor had a guardian or parent who had been in the habit of supplying his wants and making his contracts.

In this State, married women can buy, sell and make contracts and conduct any business on their own responsibility, and their husband's property would not be liable for their debt's, except in those cases where they contract a debt for necessities, and where it can be shown as such to the satisfaction of the jury, then the husband is responsible to the creditor for the liability. What are necessities in each case is for the jury to decide. The weight of opinions is that dental bills are necessities. In the matter of book accounts, to establish a bill in court in an action for money due, the books of the dentist are admissible as evidence, but what weight, depends on how methodical the entries are inserted, and if the whole journal and daybook seem to have the different items inserted properly, together with the dentist's affidavit as to the authenticity, will probably prove a claim.

There are other points I should like to have touched, but to open such subjects, and go into them as deep as I would like in order to make them clear would consume too much time, and to continue simply with definitions *ad infinitum* would be of little interest to you, and no satisfaction to myself. Gentlemen, I thank you.—*Dental Review.*

